# Coronary Function Testing: Seeking Answers for Refractory Chest Pain

**DOI:** 10.1016/j.jscai.2024.102437

**Published:** 2024-12-19

**Authors:** Connor P. Tice, Kathleen E. Kearney, John E.A. Blair

**Affiliations:** Division of Cardiology, Department of Medicine, University of Washington, Seattle, Washington

**Keywords:** coronary function testing

## Case report

A 73-year-old man with a complex coronary history was referred for evaluation of chronic angina. He had a history of acute myocardial infarction for which he underwent percutaneous coronary intervention (PCI) to his left circumflex and first diagonal arteries. This was followed by repeat PCI of the circumflex 1 month later and eventual coronary artery bypass grafting 1 year after his index acute coronary syndrome event with grafts to the first diagonal and obtuse marginal arteries, all approximately 10 years prior to this presentation. Recent hospitalization for unstable angina requiring frequent sublingual nitrate administration prompted repeat coronary angiography, which revealed a new high-grade stenosis of his patent ductus arteriosus, prompting intravascular ultrasound-guided PCI. He reported an initial improvement of symptoms, but angina returned less than a week later despite optimization of medical therapy. Given severely limiting angina despite complete revascularization, he was referred for coronary function testing (CFT) for evaluation of angina.

## Functional testing

Through CFT, a patient can be evaluated for pathology in both the epicardial vessels and microcirculation including epicardial spasm, functional myocardial bridging, endothelium-independent coronary microvascular dysfunction (CMD), microvascular spasm, and endothelial dysfunction. Information about coronary flow and resistance is aided by wiring the vessel (typically the left anterior descending [LAD] artery) with a pressure and temperature probed wire (PressureWire X, Abbott). A patient first undergoes spasm and endothelial dysfunction testing with an endothelium-dependent vasodilator, acetylcholine (ACh). A test dose of 20 μg followed by low-dose ACh (40 μg) is administered intracoronary through a guiding catheter over 30 seconds. The patient is monitored for chest pain or electrocardiogram (ECG) changes, which, if present in the absence of epicardial spasm, would indicate microvascular spasm.^1^ High-dose ACh (100 μg) is then administered intracoronary to evaluate for epicardial coronary vasospasm. Epicardial spasm is defined as a >90% transient stenosis with angina and new ischemic ECG changes.^1^ With the PressureWire X advanced across the LAD artery before administration of ACh, the hemodynamic significance of spasm was assessed using the radio of mean distal coronary artery pressure (Pd) to mean aortic pressure (Pa). Although there are no established cutoffs of ACh Pd/Pa, we feel this adds objective insights into hemodynamics in the setting of spasm testing by showing hemodynamic limitation in flow in some patients who may not have >90% transient stenosis. Nitroglycerin is then administered to reverse induced spasm before proceeding to an assessment of CMD. Adenosine, an endothelial-independent vasodilator, is used to induce hyperemia via the vasodilation of the precapillary arterioles, and coronary flow reserve (CFR) and index of microcirculatory resistance (IMR) are assessed. CFR is assessed as the ratio of hyperemic to nonhyperemic coronary flow. Coronary flow is estimated as the inverse of transit time of a 3-mL saline intracoronary bolus based on thermodilution principles. This metric assesses the ability of the coronaries to respond to the demand for increased perfusion.^2^ IMR is calculated as the ratio of hyperemic mean Pd to hyperemic flow and is used to assess for increased microvascular resistance.^2^ CMD is defined as CFR <2.0 or IMR ≥25 after adenosine administration.

**Figure 1:**
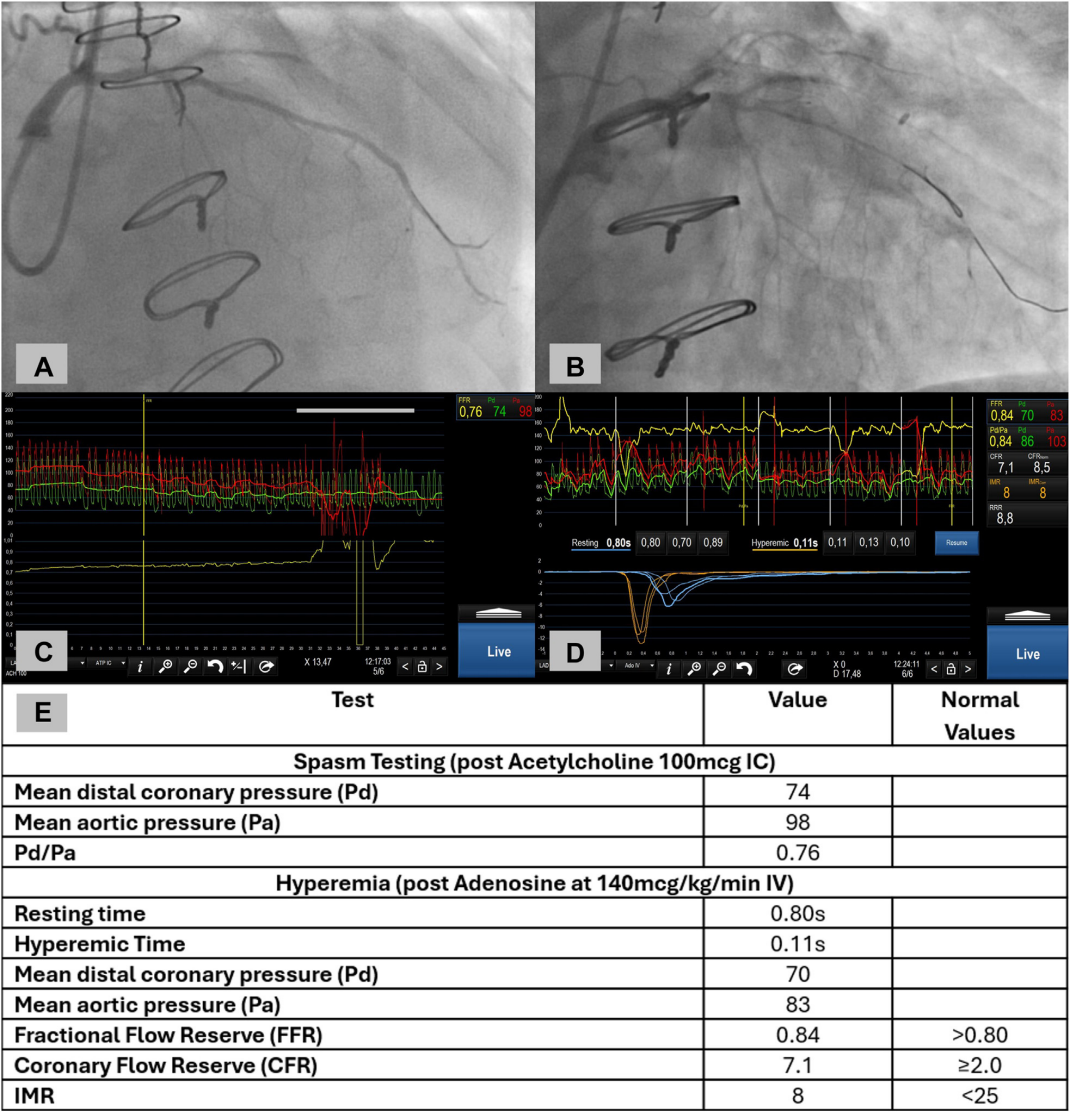
**(A) Representative still-frame from coronary angiogram; pre-acetylcholine.** (**B**) Still-frame post-acetylcholine showing diffuse spasm. The more proximal wire was used to stabilize the guide for advancement of the PressureWire X and was left in place at the time of spasm testing. (**C**) Snapshot of the nadir Pd and Pa tracings after 100 μg acetylcholine was administered. The loss of aortic signal tracing toward the end of the snapshot was during contrast injection to verify high-grade transient stenosis. The wire is stationary in the distal left anterior descending artery. (**D**) Invasive physiology with rest and hyperemia (adenosine) values to obtain FFR, thermodilution CFR and IMR showing no CMD. The 3 upper left panels display wire (green) and aortic (red) resting pressure, with the corresponding resting Pd and Pa measurements in the upper right panel. The next upper 3 panels display wire (green) and aortic (red) hyperemic pressure with the corresponding measurements of Pd and Pa as well as FFR in the upper right panel. The curves below the pressure signals represent resting (blue) and hyperemic (brown) temperature over time curves with the corresponding transit times below these curves and CFR and IMR displayed in the upper right panel. (**E**) Table of hemodynamic values including established normal values. CMD, coronary microvascular dysfunction; CFR, coronary flow reserve; FFR, fractional flow reserve; IC, intracoronary; IMR, index of microcirculatory resistance; IV, intravenous; Pa, mean aortic pressure; Pd, mean distal coronary artery pressure.

After a diagnostic coronary angiography demonstrating no obstructive coronary artery disease and no myocardial bridging, our patient underwent CFT to further evaluate the etiology of his symptoms. The patient had no microvascular spasm based on the absence of chest pain or ECG changes after a low dose of ACh; however, he had 95% diffuse spasm of the entire LAD artery with ST depression and chest pain with high-dose ACh that improved with administration of intracoronary nitroglycerin (Figure 1A, B). ACh Pd/Pa was 0.76 with high-dose ACh, likely representing severe ischemia in the spasm segment (Figure 1C, E). Finally, CFR and IMR were normal in this patient, suggesting no CMD (Figure 1D, E). Taken together, we diagnosed our patient with significant epicardial vasospasm without fixed CMD (normal CFR and IMR with adenosine). Both the non-dihydropyridine calcium channel blocker diltiazem and the third-generation nitric oxide-releasing β blocker nebivolol have been shown to be effective for vasospastic angina.^3^ As this patient was currently requiring amlodipine with well-controlled hypertension, we chose to add nebivolol. Compared to other β blockers that have been shown to worsen vasospasm, nebivolol, with its β1 selectivity, also promotes endothelial dependent vasodilation via nitric oxide pathways.^4^ On clinical follow-up, frequency and intensity of angina had significantly improved with only 1 episode of “mild” angina over 5 months postprocedure.

## Summary

CFT can provide diagnostic clarity and potential therapeutic options for patients who present with angina yet have nonobstructive coronary arteries on coronary angiography. A systemic approach to evaluate for specific causes of chest pain with CFT has been shown to improve angina.^5^ Based on our comprehensive evaluation of our patient’s chest pain, we were able to create therapeutic plan that can be tailored to treat the specific pathology involved, likely resulting in his improved symptoms.

## Pearls in Hemodynamics

• Coronary angiography, although helpful in diagnosing severe stenosis, is inadequate for determining the cause of angina, even in patients with complex coronary artery disease.
• Coronary function testing using both an endothelial-dependent vasodilator (e.g. acetylcholine) and endothelial-independent vasodilator (eg, adenosine) provides a comprehensive picture of possible explanations for angina with nonobstructive coronary arteries.
• By identifying the underlying etiology of angina through systematic functional testing, a patient’s therapeutic plan can be tailored to treat the specific pathology involved, which has been shown to improve angina over standard care.
